# Preclinical Assessment of a ^68^Ga-DOTA-Functionalized Depsipeptide as a Radiodiagnostic Infection Imaging Agent

**DOI:** 10.3390/molecules22091403

**Published:** 2017-08-24

**Authors:** Thomas Ebenhan, Botshelo Brenda Mokaleng, Jacobus Daniel Venter, Hendrik Gert Kruger, Jan Rijn Zeevaart, Mike Sathekge

**Affiliations:** 1 University of Pretoria & Steve Biko Academic Hospital, Crn Malherbe and Steve Biko Rd, Pretoria 0001, South Africa; Thomas.ebenhan@gmail.com (T.E.); tshelom@gmail.com (B.B.M.); 2 South African Medical Research Council, 1 Southpansberg Road, Pretoria 0001, South Africa; kobusventer347@gmail.com; 3 School of Health Sciences, Catalysis and Peptide Research Unit, University of KwaZulu Natal, E-block 6th Floor, Westville Campus, University Rd, Westville, Durban 3630, South Africa; kruger@ukzn.ac.za; 4 Department of Science and Technology, Preclinical Drug Development Platform, North West University, 11 Hoffman St, Potchefstroom 2520, South Africa; janrijn.zeevaart@necsa.co.za

**Keywords:** DOTA-TBIA101, depsipeptides, infection, inflammation, tuberculosis, ^68^Ga/^68^Ge generator, PET/CT imaging

## Abstract

The study assessed a radiolabeled depsipeptide conjugate (^68^Ga-DOTA-TBIA101) for its potential as an imaging agent targeting infection or infection-associated inflammation. ^68^Ga-labeled DOTA-TBIA101 imaging was performed in (NZR1) healthy rabbits; (NZR2) rabbits bearing muscular sterile inflammation and *Staphylococcus aureus* (SA) infection; and (NZR3) rabbits infected with *Mycobacterium tuberculosis* (MTB) combined with a subcutaneous scruff infection of SA in the same animal. All animals were imaged using a PET/CT scanner at 5 and 60 min post injection. Images showed elevated accumulation of ^68^Ga-DOTA-TBIA101 in the sterile muscular inflammation site (T/NT ratio = 2.6 ± 0.37 (5 min) and 2.8 ± 2.3 (60 min)) and muscles infected with MTB (T/NT ratio = 2.6 ± 0.35 (5 min) and 2.8 ± 0.16 (60 min)). The findings suggest that ^68^Ga-DOTA-TBIA101-PET/CT may detect MTB-associated inflammation, although more foundational studies need to be performed to rationalize the diagnostic value of this technique.

## 1. Introduction

Bacterial infections are some of the most challenging disease risk factors in health care, notwithstanding significant global developments in antimicrobial chemotherapy. Infection still remains a key cause of morbidity and mortality due to poor diagnosis and increased drug resistance [1]. Due to the various mechanisms of pathogenesis, early stage diagnosis is essential in the effective prevention and treatment of bacterial infections [2]. Radiological methods, such as ultrasound or computed tomography (CT) may be useful but are not always capable of differentiating septic from aseptic processes, since they can only identify anatomical changes related to late stage infections. Magnetic resonance imaging (MRI) cannot be applied in patients suffering from claustrophobia or bearing implanted medical devices. Scintigraphy imaging of infection enables the detection of the location and degree of infection in the entire body, as images are based on functional tissue changes. Infection can thus be visualized in its early stages. Approved tracers for single photon emission computed tomography (SPECT) and positron emission tomography (PET) procedures include in vitro or in vivo radiolabeled leukocytes (WBC), ^18^F-fluorodeoxyglucose (^18^F-FDG), ^67/68^Ga-citrate, and radiolabeled antibodies targeting leukocyte antigens [3]. These radiopharmaceuticals show massive variation in selectivity to diagnose infection [4,5], hence the development of bacteria-specific tracers are required, leading to more reliable tools for infection imaging. Radiolabeled antibiotics, vitamins, other biomimetics, antimicrobial peptides, and bacteriophages have been underlined as potential infection imaging agents [6,7,8,9,10].

Antimicrobial peptides (AMP) have been found to constructively bind to the bacterial membrane of Gram-positive or Gram-negative pathogens. This interaction may be useful in exploring AMPs as nuclear imaging agents for targeting bacterial infection [11]. Technetium-99m-labeled (^99m^Tc) ubiquicidin (UBI) fragments such as UBI29-41 (^99m^Tc-UBI29-41) are proven to detect bacterial infection using single-photon emission computed tomography (SPECT) in human clinical trials [12]. Although SPECT is a commonly available imaging technique, PET has numerous advantages over SPECT in terms of image resolution, sensitivity and quantification. A UBI PET ligand was, thus, verified with gallium-68 (^68^Ga), emphasized as a PET-radioisotope meeting the physiological half-life of peptides [13], and attached to the biological targeting vector (i.e., peptide bioconjugates). Chelation with bi-functional chelator molecules [14,15,16] was used to conjugate UBI to 1,4,7-triazacyclononane-1,4,7-triacetic acid (NOTA) (^68^Ga-NOTA-UBI). This allowed us to develop a ^68^Ga radiolabeling procedure [17], followed by the successful preclinical evaluation of this radiotracer as a selective imaging agent that targets muscular bacterial manifestation in a rabbit model. It has also been indicated that the conjugation of AMP with a bifunctional chelator moiety may not compromise the peptide capability in differentiating between inflammation and infection [18].

A recent study proved that ^68^Ga-1,4,7,10-tetraazacyclododecane-1,4,7,10-tetraacetic acid (DOTA)-peptides can be used for infection imaging [19]. This led to the conclusion that particular fragments of antimicrobial peptides are matching vector molecules to allow conjugation with DOTA. This bioconjugate can be subsequently radiolabeled with radiometal-isotopes such as ^68^Ga, and used for PET image acquisition [20]. Depsipeptide derivatives have emerged as promising leading compounds for new drug discovery with superior activity against multidrug-resistant bacteria [21]. It has been shown that the antibacterial and hemolytic activities of antimicrobial peptide depends on charge, size, secondary structure, hydrophobicity, and amphipathicity, which have a major effect on their ability to depolarize the bacterial membrane [22]. The presence of hydrophobic amino acids in the depsipeptide sequence is crucial for antibacterial activity and position 3 in the sequence is tolerable to amino acid changes by introduction of neutral and hydrophobic amino acid without losing antibacterial potency; depsipeptides also induce positive inotropic and negative chronotropic cellular effects [23].

**Figure 1:**
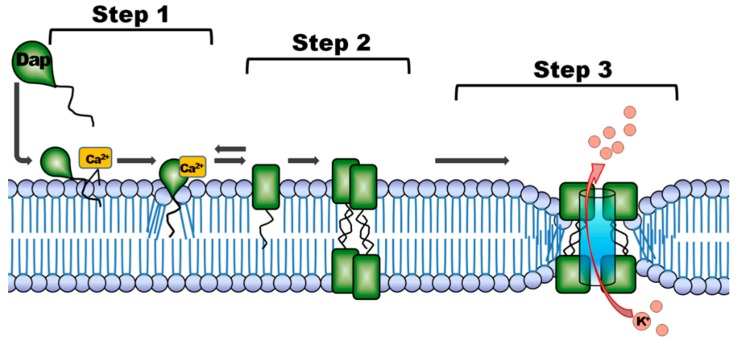
Potential antimicrobial mechanism suggested for depsipeptides. The postulated mechanism for daptomycin (Dap) is adapted from Steenbergen et al., 2005 [24]: step 1: binding to the cytoplasmic membrane; step 2: oligomerization and membrane disorientation, and step 3: release of intracellular ions followed by cell death.

The postulated antimicrobial mechanism of action of depsipeptides (Figure 1) may be explained as follows [24]: Step (1) interaction and association with the bacterial cell envelope; step (2) oligomerization, pore formation and disruption of the bacterial membrane; and step (3) release of intracellular K^+^ -ions leading to rapid cell death. The mechanism requires the presence of Ca^2+^ ions, which play a role in binding to Cl^−^-ions within the lipid layer [25]. During the binding to the bacterial membrane the peptide undergoes two Ca^2+^-dependent structural transitions, which are desired targeting properties of an imaging agent for PET. Moreover the presence of negatively-charged lipids will allow depsipeptides to further insert into and perturb bilayer membranes with acidic character [26]. The understanding of this mechanism may suggest depsipeptides as a novel class of AMP for potential use as radiodiagnostic agents for imaging of infection.

**Figure 2:**
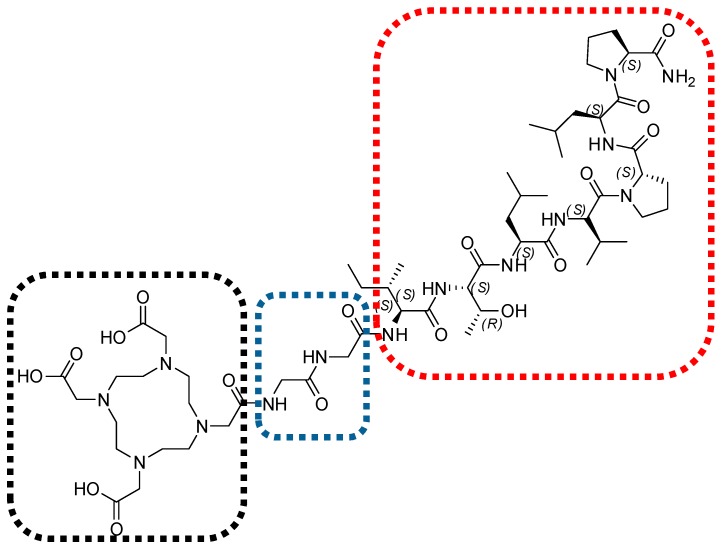
Chemical structure of DOTA-TBIA101. The depsipeptide NH_2_-l-Proline-l-Leucine-l-Proline-l-Valine-l-Leucine-l-Thronine-l-Isolleucine-OH (TBIA101, red-dotted area) is linked via bi-l-Glycine (blue-dotted area) with the bifunctional chelator molecule 1,4,7,10-tetraazacyclododecane-1,4,7,10-tetraacetic acid (DOTA, black-dotted area).

Our pilot study reports the depsipeptide synthesis of NH_2_-l-Proline-l-Leucine-l-Proline-l-Valine-l-Leucine-l-Thronine-l-Isolleucine-OH (TBIA101), and successful DOTA conjugation using a bi-l-Glycine-linker (Figure 2) which was introduced to prevent DOTA from affecting any hydrogen bonding with the peptides backbone that could influence the natural configuration of TBIA101. Additionally, we succeeded in radiolabeling DOTA-TBIA101 with ^68^Ga, which allowed for PET/CT imaging in targeting myositis in *Escherichia coli* infected mice [27]; however, no further preclinical evaluation was possible using this model.

In this study ^68^Ga-DOTA-TBIA101 was assessed for its bacterial selectivity, sensitivity, and specificity using PET imaging to study staphylococcal and mycobacterial infections against sterile inflammation in different rabbit myositis models.

## 2. Results

### 2.1. ^68^Ga-Radiolabeling and Tracer Formulation

In preparation for animal use, radiochemical yields of 68 ± 18% (*n* = 12) were achieved with a specific activity of 13 ± 6 GBq/µmol. High product desorption rates of 75–98% ^68^Ga-DOTA-TBIA101 were achieved from a C18-SepPak SPE-matrix by using 50% ethanolic saline solution. The total recovered yield (not decay-corrected) for injection was 482 ± 183 MBq (374–796 MBq, *n* = 12), sufficient activity to inject two animals within 45 min. The radiochemical purity of ^68^Ga-DOTA-TBIA101 was >98% with residual amounts of ethanol ranging 1.0–3.5%.

### 2.2. ^68^Ga-DOTA-TBIA101 Biodistribution in Healthy Animals

**Figure 3:**
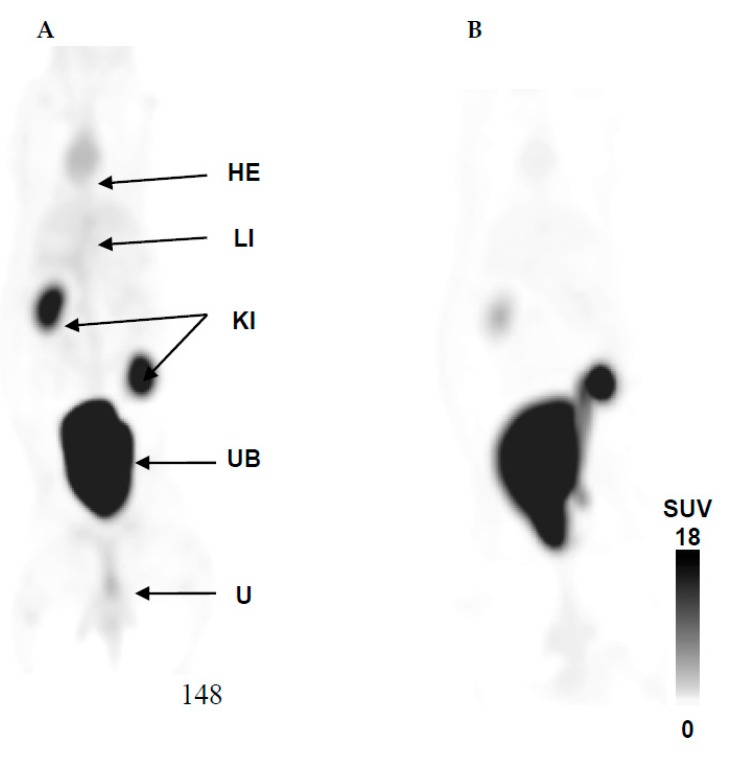
Healthy biodistribution of ^68^Ga-DOTA-TBIA101 at (**A**) 5 min and (**B**) 60 min post-tracer injection. Maximum intensity projection (MIP) PET/CT images show the expected biodistribution in heart (HE), liver (LI), and strong excretion represented by activity in kidneys (KI), urinary bladder (UB), and urine.

The radioactivity uptake in organs and tissues was expressed as %ID/g and SUV. Quantification of the biodistribution of ^68^Ga-DOTA-TBIA101 of NZR1 (comparing images acquired at 5 and 60 min post tracer injection) is presented in Table 1, while the PET/CT imaging is illustrated in Figure 3.

Except for the urinary bladder, the biodistribution of all organs showed early-onset uptake at 5 min followed by diminishing levels of radioactivity at 60 min post injection (*p* ≤ 0.05). The stomach showed the lowest overall uptake of the tracer of <0.005 %ID/g. Similarly, the organ/tissue concentration (SUV) of ^68^Ga-DOTA-TBIA101 was reduced at 60 min post injection as represented in Table 1. The highest tracer SUVs were found in the kidneys and bladder, suggesting that major amounts of activity are excreted via the renal route. Significant tracer washout was determined for spleen, lung, and the triceps muscle tissue. The activity reduction in other organs/tissues was less significant (*p* ≥ 0.1; i.e., heart muscle, liver, kidneys, stomach, and quadriceps muscle). The maximum intensity projection in Figure 3 confirms the results of the image guided SUV quantification; apart from the renal excretion, the heart muscle and liver are clearly visualized (Figure 3A) and show tracer washout over time (Figure 3B). No notable pulmonary and abdominal uptake was observed at 60 min post-tracer injection.

### 2.3. ^68^Ga-DOTA-TBIA101 Selectivity

**Figure 4:**
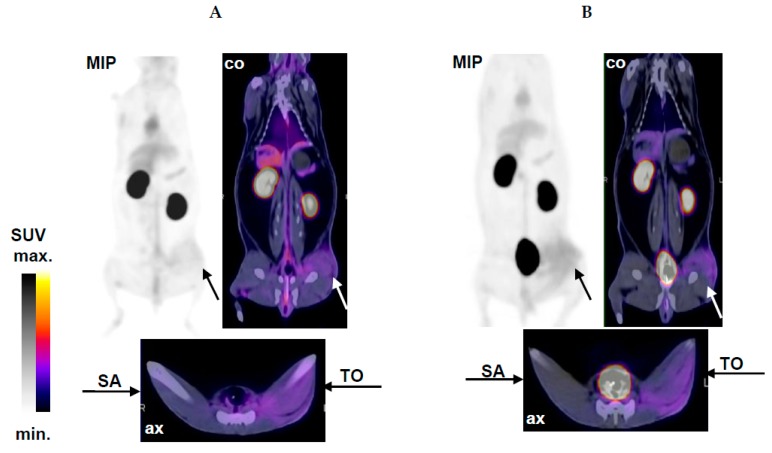
Selective imaging of sterile inflammation and SA infection in NZR2 at (**A**) 5 min and (**B**) 60 min after injection of ^68^Ga-DOTA-TBIA101. Arrows highlight the site of sterile inflammation after TO treatment and the contralateral staphylococcal infection site (SA) in a representative midplane-coronal (co) and axial (ax) PET/CT slice and in maximum intensity projection (MIP) PET image.

Localization of sterile inflammation and inflammation associated with acute staphylococcal infection by ^68^Ga-DOTA-TBIA101-PET/CT imaging was carried out in NZR2 following tracer injection at 5 and 60 min, as represented in Figure 4. The T/NT ratios for the relevant muscle tissues are summarized in Table 2.

Aside from the expected biodistribution, significant tracer uptake in the thigh muscle tissue challenged by a turpentine oil injection was observed. However; only a faint uptake was determined in the site of staphylococcal infection. Images acquired at 60 min showed a more pronounced signal-to-noise-ratio over early-on images acquired at 5 min post tracer injection. PET/CT images in Figure 4 clearly visualize the inflamed tissue in the hind muscle (TO). The CT shows a swollen muscle which was confirmed prior to image acquisition by palpation and warmness of the area. PET images reported radioactivity uptake occurring in a dispersed manner in the left hind muscle (i.e., intra-, inter-, and some extramuscular tissue). The contralateral muscle tissue (healthy) appeared normal and showed no notable (unspecific) tracer accumulation.

### 2.4. PET/CT Imaging of Mycobacterial Infection with ^68^Ga-DOTA-TBIA101

**Figure 5:**
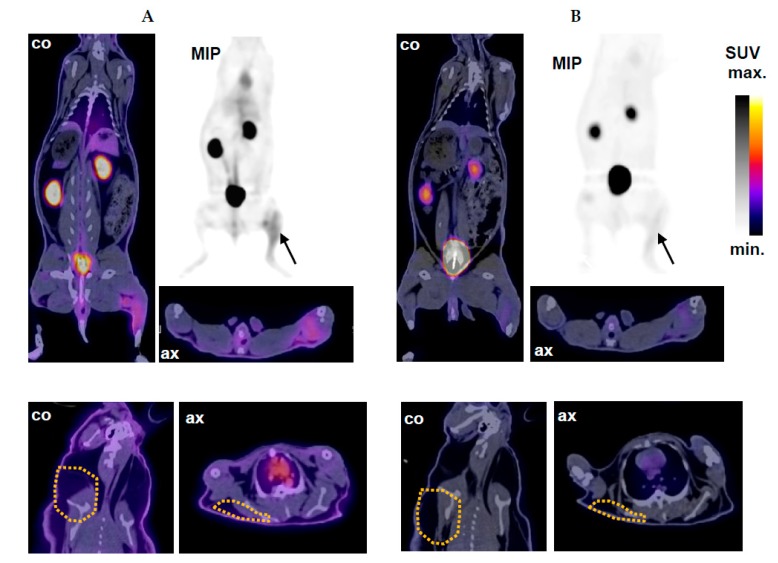
PET/CT imaging of NZR3 at (**A**) 5 min and (**B**) 60 min after injection of ^68^Ga-DOTA-TBIA101. Top panel: the MTB infection was successfully delineated by the tracer. Arrows highlight the infection site in a representative midplane-coronal (co) and axial (ax) PET/CT slice and in a maximum intensity projection (MIP) PET image. Bottom panel: A viable staphylococcal infection site in the scruff area (yellow-dotted lines) was not localized by PET/CT imaging as presented in coronal (co) and axial (ax) PET/CT slices.

^68^Ga-DOTA-TBIA101-PET/CT imaging in NZR3 detected a mycobacterial thigh infection combined with a subcutaneous SA infection in the scruff area following tracer injection at 5 and 60 min (Figure 5). Elevated ^68^Ga-DOTA-TBIA101 uptake was observed in NZR3 bearing a thigh infection of MTB. The maximum intensity projection display tracer uptake in the MTB-infected site, appeared dispersed over the entire hind leg (indicated by arrows); images acquired at 5 min showed a fair delineation of the MTB-challenged area whereas the visualization thereof became less favorable at 60 min. The uninfected contralateral muscle tissue appeared normal with no notable tracer accumulation. Visualization of the SA infection was challenged by minute tracer uptake in the challenged scruff area (Figure 5, bottom panel).

The PET/CT image-guided SUV quantification calculated a three-fold higher tracer concentration in the MTB infection site at 5 min post injection. The MTB/uninfected ratio remained the same at 60 min post injection. The expected biodistribution was insignificantly different compared to NZR1, except for higher activity levels found in the kidneys and bladder (*p* = 0.11). In addition, SA infected scruff tissue showed insignificant uptake compared to the contralateral reference tissue. The tracer accumulation was not pronounced enough (SUV of 0.95–1.55) to delineate the infection site. The T/NT ratios for the infected/reference tissues are summarized in Table 2.

### 2.5. PET/CT Image Quantification

An overview is given of the T/NT ratio calculation based on the mean SUV quantification for all NZR groups (Table 2). Significant differences were observed for the T/NT ratios in NZR2 (TO-treated muscle tissue) and NZR3 (MTB-infected muscle tissue) compared to NRZ1 (healthy muscle tissue). No significant increases in the T/NT ratios (*p* ≤ 0.1) were calculated when comparing PET/CT images at 5 min with 60 min for NRZ1, NRZ2, or NZR3.

### 2.6. Histopathological Examination

The macroscopic and microscopic examinations confirmed that all rabbits showed a significant presence of microorganisms in the relevant tissues (muscle- and adjacent tissue as well as subcutaneous tissue); the cultured CFU from homogenized tissue was identified as SA or MTB by bacteriology. As expected, TO-treatment caused significant myositis including slight evidence of fibrosis and tissue necrosis. All reference tissues and organ tissue from infected animals (healthy muscle, heart, liver, spleen, lung, and hilar lymph node) were free of microorganisms and showed no signs of inflammation.

## 3. Discussion

Infection and sterile inflammation can be detected with nuclear medicine techniques at early stages, though current tracers are unable to differentiate between infection and inflammation progressions. The development of new infection-specific agent would greatly progress the capacity to detect, localize, and quantify infection. Generally, antimicrobial peptides can be considered to be more specific than tracers that target host-activated immune cells such as radiolabeled leukocytes (considered the standard method to date). The present study was commenced with ^68^Ga-DOTA-TBIA101 to investigate it as a potential infection imaging agent. The tracer was evaluated for its practicability to differentiate between infection and inflammation by using healthy rabbits and different infection models. The study was motivated by the accomplished usage of other AMP in targeting infection in vivo [11]. Other ^68^Ga-DOTA-peptide also succeeded in oncologic diagnostics [28,29]. With the improved availability of commercial ^68^Ge/^68^Ga generators, other potential ^68^Ga-labeled radiopharmaceuticals were considered as infection imaging agents, such as ^68^Ga-citrate, ^68^Ga-apo-transferrin and ^68^Ga-siderophores.

### 3.1. Biodistribution

Labeling and purification was successfully achieved for ^68^Ga-DOTA-TBIA101, showing percentile labeling efficiency comparable to other DOTA-conjugated peptides [30,31]. Healthy rabbits showed avid biodistribution of ^68^Ga-DOTA-TBIA101 within 60 min, with rapid renal excretion. In general, this is an advantageous result, as it reduces the radiation exposure for a patient, but will make the detection of renal infection or infections adjacent to the kidney and urinary bladder challenging. A similar study performed using ^68^Ga-DOTA-VAP-PEG-P2 showed rapid renal excretion from 5 to 120 min [32]. Apart from kidney, liver, and bladder, only minimal activity was found in heart, spleen, lung, stomach, thigh, and triceps muscles, which is a desired property for a radiotracer. These findings indicate that ^68^Ga-DOTA-TBIA101 biodistribution may not interfere with non-targeted organs. Higher liver uptake was detected than in a previous described study using ^68^Ga-DOTA-nitroimidazole, where the authors reasoned that the hepatic tracer accumulation was due to high lipophilicity [33]. The high hydrophobicity of some AMPs can be considered beneficial, as it may enable rapid renal excretion [32], while lipophilicity is a requirement for a tracer to enter cell membrane and blood-brain barrier.

### 3.2. Selectivity

Contrary to our expectation, elevated uptake of ^68^Ga-DOTA-TBIA101 was found in the turpentine-treated muscle tissue at both time points, and only faint uptake in muscle tissue inoculated with SA. This result suggests a lacking bacterial selectivity for DOTA-TBIA101; however, it is supported by a previous study showing that image acquisition, as early as 5 min post injection, was sufficient to detect the sterile inflammation, which is reported for other tracers [34]. In contrast, a similar study performing ^68^Ga-NOTA-UBI-PET/CT imaging reported no uptake in turpentine-inflamed muscle and significant uptake in staphylococcal-infected muscle [20] ^68^Ga-DOTA-TBIA101-PET/CT may be unsuccessful in detecting a mild myositis caused by staphylococcal inoculation; the infected muscle showed T/NT ratios that were in the same range as in non-targeted muscle (triceps or quadriceps). This suggests that the tracer is not sensitive for SA or SA-associated inflammation (in this study design) which did not meet the expectation. Our previous study reported ^68^Ga-DOTA-TBIA101 uptake in an *Escherichia coli*-infected muscle [27], which was significant to delineate the infection site. We can currently safely conclude that this was a representative image of the infection-associated inflammation. ^68^Ga-transferrin has also been indicated to detect inflammation associated with both Gram-negative and Gram-positive bacteria [35].

Generally, AMPs have different biological activity profiles; however, most of them act directly on the bacterial cell membrane. Several studies have reported that AMPs can interfere with the cellular properties and metabolic functions [36]. The effect on the mode of action for modified depsipeptides is not fully understood; when unmodified, they are active against an array of bacteria and follow a unique binding mechanism (Figure 1) and, upon internalization, it is known that depsipeptides inhibit cellular function by binding to DNA and RNA [37]. This study made use of a linear peptide sequence instead of the more natural cyclic form. It is quite conceivable that, by deviating from the cyclic peptide formation of TBIA101 to address the radiolabeling, the peptide selectivity was crucially compromised. In addition, even though the tracer design considered separating the ^68^Ga-chelator (DOTA) from the targeting entity, it cannot be assumed that some structural, steric hindrance occurred; different linker molecules (e.g., polyethylenglycol (PEG)) might be more suitable and will improve the molecule. Future approaches to functionalize cyclic depsipeptides should consider introducing covalently-bound radioisotopes such as ^18^F or ^123/125^I, as such radioisotopes would negate the addition of a chelator molecule. Structurally, lipodepsipeptide-derived compounds could rather be the preferred AMP class (i.e., daptomycin) as they incorporate a lipophilic “tail” which is a highly important structure in facilitating the initial step of binding to the bacterial membrane [38].

### 3.3. Mycobacterial Imaging

In general, the in vivo results indicated that the infection is detectable within three days post mycobacterial inoculation and tends to extinguish over time. Depsipeptides have been found to strongly inhibit the monocyte-triggered immune response to in vitro MTB infection without affecting growth in broth culture. The infected animals retained radioactivity in their mycobacteria-infected thighs; elevated uptake, but without a substantial increase in the T/NT ratio over time (opposite to the expectation). This scenario is not suggestive for direct tracer binding to MTB in vivo. In the same infection model (i.e., NZR3), increasing uptake of another radiolabeled AMP (^68^Ga-UBI) between 5 and 60 min post injection validated the staphylococcal presence in the scruff and the mycobacterial load in the thigh muscle [39]. This suggests that ^68^Ga-DOTA-TBIA101-PET/CT imaging rather represented the inflammation in connection with the MTB infection than the mycobacterial burden to the muscle tissue. In a similar tracer assessment, PET/CT imaging using ^68^Ga-DOTAVAP-P1 was carried out two days post cocci injection with a two-fold higher bacterial load [19]. The infected muscle to background ratio of ^68^Ga-DOTA-TBIA101 (2.3 ± 0.3) was in the same range with the latter study (2.3 ± 0.7) and lower compared to ^18^F-FDG (3.1 ± 0.6). Between the T/NT ratios of NZR3 to NZR1, a significant difference in tracer uptake, due to myositis, was determined (Table 2; *p* < 0.01). Despite the lacking selectivity towards imaging bacteria directly (which ^68^Ga-UBI-PET/CT is competent for) the results on DOTA-TBIA101 demonstrate that this radiotracer yields high T/NT ratios for inflammation, and similar ratios in inflammation are associated with mycobacterial infection. Again, this imaging technique cannot be considered bacteria-selective, but is nevertheless sensitive enough to localize muscular MTB. Although other researchers have turned their attention to complexing ^68^Ga with peptides in attempts to develop more specific imaging agents, ^68^Ga-DOTA-TBIA101 still experience limited specificity, similar to a reported PET study performing infection imaging with ^68^Ga-citrate [40]. However, ^68^Ga-DOTAVAP-P1-bringing along similar diagnostic difficulties-has been indicated to be useful for imaging early-onset inflammation and infection in healing bones [41]. Similarly, the reported study confirmed using ^68^Ga-DOTAVAP-P1 as an infection imaging agent [19].

### 3.4. Clinical Relevance and Limitations

At present, no approaches to radiolabeling depsipeptides-derivative compounds have been reported using medicinal radioisotopes for subsequent validation in imaging of infection or inflammation. Therefore, the benefits of ^68^Ga-DOTA-TBIA101 harboring a peptide-based vector display a new aspect to infection imaging compared to tracers such as ^18^F-FDG, radiolabeled white blood cells or ^67/68^Ga-citrate. This tracer can be produced in-house (cyclotron independent) using the on-demand radioactivity of a commercial ^68^Ge/^68^Ga generator. It is a simplified, cost-efficient labeling approach and potentially offers shorter imaging times with less radioactivity burden (favorable dosimetry). The authors introduced a novel class of AMPs in this study; however the assessment proved the hypothesis mistaken towards development of a selective agent for infection imaging. The mycobacterial infection detected by ^68^Ga-DOTA-TBIA101 requires better understanding and cannot be considered selective. The usefulness of depsipeptide (or more appropriate depsipeptide derivatives) is currently limited as an infection imaging agent in nuclear medicine and should be re-evaluated using an optimized chemical bioconjugate.

## 4. Materials and Methods

This animal study was conducted in accordance with guidelines prescribed by the South African Medical Research Council (MRC), and ethical approval was granted by the Ethics Committee for Research on Animals (ECRA No.: 03/12) at the University of Pretoria.

### 4.1. Material

DOTA-TBIA101 was kindly provided by the Catalysis and Peptide Research Unit, University of KwaZulu-Natal (Durban, South Africa). If not stated otherwise, reagents and biochemicals were purchased from Sigma Aldrich (Kempton Park, South Africa) in the highest available grade and used without further refinements. *Staphylococcus aureus 25923* (SA) and *Mycobacterium tuberculosis H37Rv* (MTB) were kindly provided by the Medical Research Council Tuberculosis Intervention Unit (Pretoria, South Africa). Certified 10 mL borosilicate glass vials for generator elution and radiolabeling were purchased from AEC-Amersham (Johannesburg, South Africa).

### 4.2. ^68^Ga-DOTA-TBIA101 Radiolabeling and Formulation

Radiolabeling of ^68^Ga-DOTA-TBIA101 was performed as described [27]; briefly, ^68^Ga-GaCl_3_ was yielded from a 1850 MBq SnO_2_-based ^68^Ge/^68^Ga-generator (iThembaLABS, Somerset West, South Africa) by eluate fractionation. DOTA-TBIA101 was labeled at >90 °C for 15 min (pH > 4) in the presence of 1 or 2 mL sodium acetate trihydrate-buffered ^68^Ga-GaCl_3_ (740–1850 MBq; *n* = 12). Solid-phase extraction (SPE) purification was carried out using SepPak C18-light cartridge units (Microsep, Johannesburg, South Africa). The pure radiolabeled product was yielded in a 50% ethanolic saline solution. For the preclinical use ^68^Ga-DOTA-TBIA101 was formulated in 10 mL sterile saline solution to reduce the ethanol content to ≤4%. The final product was aseptically filtered and adjusted to a physiologic pH; a bolus of 4–6 mL was dispensed for the intravenous tracer administration.

### 4.3. Study Design, Animal Preparation, and Tracer Administration

The study engaged 14 New Zealand White rabbits (NZR: male, 18–24 months, body weight: 2.9–4.2 kg). The imaging study was carried out in the following groups: NZR1) tracer biodistribution in healthy animals (NZR1; *n* = 3), NZR2) tracer selectivity in animals bearing a staphylococcal thigh muscle inoculum (1 × 10^8^ cfu) paired with an injection of 0.5 mL turpentine oil (TO) into the contralateral thigh muscle (NZR2; *n* = 4). In addition, ^68^Ga-DOTA-TBIA101 sensitivity was tested targeting mycobacterial infection following a thigh muscle inoculum with 1 × 10^8^ CFU of MTB paired with a subcutaneous inoculum (1 × 10^8^ cfu) of SA (NZR3; *n* = 7) in the same animal. Based on former tracer assessments all imaging procedures were carried out three days after inoculation [20]. Prior to PET/CT imaging animals were immobilized with ketamine/medetomidine (15/0.5 mg/kg) (Xylavet, Kempton Park, Johannesburg, South Africa) and was maintained by propofol/thiopental (10/25 mg/kg) (Fresenius Kabi, Midrand, Johannesburg, South Africa) administration via an ear catheter. The left ear vein was catheterized to administer a 3.5–6 mL ^68^Ga-DOTA-TBIA101 bolus dose of 42 ± 4 MBq/kg, 53 ± 6 MBq/kg and 64 ± 5 MBq/kg to NZR1, NRZ2, and NZR3, respectively.

### 4.4. PET/CT Imaging

All image acquisitions, reconstructions, and analyses were performed at the Department of Nuclear Medicine, Steve Biko Academic Hospital (University of Pretoria, South Africa) using a clinical tomograph (Biograph40 True Point PET/CT, Siemens AG, Erlangen, Germany) with licensed Siemens software (*syngo* E.soft 2.0.21; Siemens AG, Erlangen, Germany). Dual-time point PET/CT was conducted at 5 and 60 min post-intravenous tracer injection with rabbits placed in a supine position. All images were acquired with a 4 min emission scan for each of the 4–5 bed positions (matrix size 512 × 512). Reconstruction of images with and without CT-based attenuation correction was done using ordered subset expectation maximization (OSEM) to yield axial, sagittal, and coronal slices. Qualitative evaluation was followed by semi-quantification by manually drawing three-dimensional volume of interests (VOI) regions including the whole body, background, a reference areal and organs, such as bladder, kidneys, lungs, liver, stomach, spleen, heart, the healthy triceps muscle, and the infected-or inflamed hind muscle tissues. The activity accumulation and the sizes selected were to match to any pathology detected on CT. The biodistribution of the tracer is represented for the two time points by percentage injected dose per gram organ (%ID/g) and the ^68^Ga-DOTA-TBIA101 tissue concentration is represented by the calculation of an average uptake value (SUV) expressed from the same area (i.e., normalized uptake values—the maximum value determines the peak concentration in the selected VOI; mean values reflect the overall tracer concentration). Subsequently, target-to-non-target (T/NT) ratios were calculated from the SUV to compare animals of all groups.

### 4.5. Bacteriology and Histopathology

All inflamed and/or infected sites were evaluated post mortem for gross tissue pathology followed by histopathological and microbiological examination. Samples of 10% PBS-buffered formalin-fixed tissues were embedded in paraffin as 0.5 μm slices. Other parts of the same tissues were homogenized and plated on appropriate agar plates for bacterial recovery. Biochemical and immune-histochemical staining included hematoxylin eosin (HE) and Ziehl-Neelsen (ZN), followed by examination by light microscopy to confirm the bacterial manifestation in the respective tissue samples.

### 4.6. Biocontaminant Prevention

The MTB strain used in this study is a bio-safety level 3 (BSL-3) type organism. To prevent biocontamination of the environment, animals were kept in individual ventilated cages in a BSL-3 filtered system to protect personnel in contagious areas. For personnel safety, personal protective equipment (PPE) was worn, which included HEPA-filtered and powered full head respirator, laboratory coats, gloves and boots. All waste and used PPE was chemically sterilized with 10% formalin before being heat sealed and discarded according to the general standard operation procedure for biohazard waste management.

### 4.7. Statistical Analysis

If not stated otherwise, the average biodistribution or activity concentration for organs or tissues were expressed as mean and standard deviation (SD) and/or standard error of mean (SEM) using Microsoft Office Excel 2010 software (Microsoft, Frankfurt, Germany). The significance of two mean values was calculated by paired or unpaired Student’s *t*-test. The levels of significance were established at *p* < 0.1 (*), >0.05 (**), and <0.001 (***).

## 5. Conclusions

^68^Ga-DOTA-TBIA101-PET/CT imaging delineated TO-induced sterile inflammation and myositis caused by mycobacterial infection from normal reference tissue in rabbit infection models. Unexpectedly, no tracer selectivity occurred towards deciphering tissue bacteremia from infection-associated inflammation. The radiopharmaceutical showed no adverse effect upon administration, but it remains questionable whether the structural design was appropriate to match the aim for sensitive and selective bacterial imaging. Further optimizations on the chemical structure of DOTA-TBIA101 are required before the usefulness of ^68^Ga-DOTA-TBIA101-PET/CT imaging can be addressed. A more conserved or separated targeting motif of this depsipeptide and a direct radiolabel with ^18^F may achieve successful differentiation of bacteria from sterile inflammation.

## Figures and Tables

**Table 1:** Organ and tissue biodistribution (%ID/g) and concentration (SUV) of ^68^Ga-DOTA-TBIA101 in healthy New Zealand White rabbits (NZR1).

	Activity Biodistribution (%ID/g) ^#^		Activity Concentration (SUV) ^#^	
Organ or Tissue	5 min	60 min	5 min	60 min
Heart/Blood Pool	0.053 ± 0.003	0.028 ± 0.003 ***	0.35 ± 0.15	0.22 ± 0.06
Liver	0.057 ± 0.007	0.017 ± 0.002 ***	0.35 ± 0.03	0.37 ± 0.01
Spleen	0.021 ± 0.0.03	0.014 ± 0.001 **	0.63 ± 0.01	0.30 ± 0.01 ***
Urinary Bladder	1.32 ± 0.15	1.24 ± 0.002	9.2 ± 7.7	14.2 ± 1.9
Kidney (L/R)	0.16 ± 0.05	0.096 ± 0.035 **	2.5 ± 2.4	2.8 ± 3.1
Lung (L/R)	0.028 ± 0.002	0.014 ± 0.003 ***	0.26 ± 0.04	0.13 ± 0.02 **
Stomach	0.004 ± 0.001	0.003 ± 0.001	0.09 ± 0.01	0.09 ± 0.04
Quadriceps (R)	0.011 ± 0.002	0.003 ± 0.001 ***	0.24 ± 0.10	0.09 ± 0.02
Triceps (L)	0.009 ± 0.002	0.003 ± 0.001 **	0.24 ± 0.04	0.11 ± 0.01 *

(^#^) Values expressed as mean (± SD) N = 3 animals. (R) right site; (L) left site; (L/R) mean value of left and right side. Two-way Student’s *t*-test analysis comparing SUV of 5 min with 60 min returned a *p* value of ≤0.1 (*), ≤0.05 (**) and ≤0.01 (***).

**Table 2:** Summary of the quantitative analysis of ^68^Ga-DOTA-TBIA101 uptake in muscle tissue (group NZR1-3).

T/NT Ratio (SUV Based)	Image Acquisition Postinjection ^$^	
	5 min	60 min
NZR1: Thigh (L/R)/Triceps (L/R)	1.2 ± 0.11	1.2 ± 0.24
NZR2: Thigh (SA)/Triceps (L/R)	1.0 ± 0.02	1.2 ± 0.15
NZR2: Thigh (TO)/Triceps (L/R)	2.6 ± 0.37 ***	2.8 ± 2.3 **
NZR3: Thigh (MTB)/Triceps (L/R)	2.6 ± 0.35 ***	2.8 ± 0.16 ***
NZR3: Thigh (MTB)/Thigh (L)	2.1 ± 0.26 ***	2.0 ± 0.31 ***
NZR3: Scruff (SA)/Scruff (R)	1.1 ± 0.12	1.3 ± 0.35

^$^ Values represent mean (± SD) N 3–7 animals; (L/R) mean value of left and right side. *p*-value of ≤0.05 (**) and ≤0.01 (***) comparing healthy with respective values in MTb-infected animals, Two-way Student’s *t*-test analysis comparing T/NT ratios 5 min and 60 min of NZR2 and 3 to NZR1 returning *p* values of ≤0.05 were considered significant.
